# Multicentric Study on the Diagnostic of Neuromuscular Diseases in Children with High Creatinine Phosphokinase Levels

**DOI:** 10.3390/children11121462

**Published:** 2024-11-29

**Authors:** Cláudia Monteiro, Cristina Garrido, Ângela Pereira, Andreia Dias, Mariana Costa, Catarina Magalhães, Manuela Ferreira, Miguel Costa, Manuela Santos

**Affiliations:** 1 Pediatrics Service, Centro Hospitalar Tâmega e Sousa, Avenida do Hospital Padre Américo 210, 4564-007 Guilhufe, Portugal; 2 Department of Neuropediatrics, Centro Materno-Infantil do Norte, Centro Hospitalar Universitário de Santo António (CHUdSA), Largo da Maternidade de Júlio Dinis 45, 4050-651 Porto, Portugal; 3 Hospital Braga, R. das Sete Fontes, 4710-243 Braga, Portugal; 4 Centro Hospitalar Trás-os-Montes Alto Douro, Avenida da Noruega Lordelo, 5000-508 Vila Real, Portugal; 5 Hospital de Santa Luzia, Estr. de Santa Luzia, 4900-408 Viana do Castelo, Portugal; mariana.costa@ulsam.min-saude.pt; 6 Hospital Senhora da Oliveira, R. dos Cutileiros 114, 4835-044 Creixomil, Portugal; 7 Unidade Hospitalar de Bragança, Av. Abade de Baçal SN, 5301-852 Bragança, Portugal; 8 Centro Hospitalar de Entre Douro e Vouga, R. Dr. Cândido Pinho 5, 4520-211 Santa Maria da Feira, Portugal

**Keywords:** neuromuscular diseases, creatine phosphokinase (CK), screening, dystrophinopathies

## Abstract

Background: Neuromuscular diseases (NMDs) are rare, predominantly hereditary, with progressive course disorders. Furthermore, diagnosis can be delayed by years after symptoms emerge, resulting in missed opportunities for modifying disease progression, specific therapeutic approaches, and counseling. Some NMDs have high levels of creatine phosphokinase (CK). Thus, its measurement can indicate an NMD associated with muscle involvement. Objectives: We aimed to identify myopathies and muscular dystrophies through elevated CK levels for early detection of these disorders. Methods: A prospective, observational, and analytical study of children and teenagers showing high levels of CK, showing mild symptoms, or who were asymptomatic with elevation of transaminases from all pediatric units in the north of Portugal was performed. All diagnosed patients were referred to our Center for Neuromuscular Diseases. Additionally, CK level confirmation, clinical examination, and investigation were performed according to best-practice clinical guidelines. Results: We found 33 patients from 8/12 pediatric units. A diagnosis with implications for care measures and treatment was performed in half of the patients. A total of 30% presented an NMD diagnosis. Dystrophinopathies represented the largest group (21%). Conclusions: Therefore, NMDs should be considered in children and teenagers with high CK levels, even those with mild symptoms. Screening for CK elevation should be used to promote an earlier diagnosis of many NMDs.

## 1. Introduction

Neuromuscular diseases (NMDs) are a broadly defined group of disorders that involve injury or dysfunction of peripheral nerves or muscles [1]. About 90% of NMDs are classified as rare diseases, showing prevalence rates between 1 and 10 per 100,000 population [2]; however, they represent one of the main causes of mortality and disability throughout life in children and adults. NMDs have many etiologies, which are mainly genetic; some are acquired [3,4]. In addition, NMDs encompass several syndromes with diverse phenotypes, but with common underlying symptoms, namely progressive muscle weakness and atrophy, often leading to dysphagia and respiratory or cardiac failure, reducing the patient’s autonomy [5,6].

High levels of creatine phosphokinase (CK) are found in many muscular dystrophies (MD) and myopathies, namely metabolic [7,8]. Some studies have shown that serum CK concentration is increased in children with MD before the appearance of any clinical signs of disease [9].

An earlier diagnosis of NMD would facilitate quicker access to health interventions, including multidisciplinary care and specific treatment for some of them, for instance, Duchenne Dystrophy [10]. Accurate and early detection of metabolic myopathies allows for timely counseling to prevent metabolic crises and helps in discovering therapeutic interventions to reduce symptoms [11]. Furthermore, a delay in diagnosing precludes genetic counseling and may put families at risk of having a second affected child [12].

Here, we report the results of a survey in the North of Portugal that relates high CK levels in children and adolescents with the early detection of muscular dystrophies and myopathies. In addition, this study aimed to raise awareness about the importance of early diagnosis of these diseases.

## 2. Materials and Methods

### 2.1. Study Design

A prospective, observational, and analytical study from the North of Portugal was performed between the 1 May 2018 and the 30 April 2020. Neuropediatricians from the Reference Center for Neuromuscular Diseases performed an overview of the value of high CK levels for the diagnosis of NMD and presented the project in all adherent pediatric departments.

Were included children and teenagers between 1 month and 18 years who presented high CK levels (at least 1.5 times the regular values) with mild symptoms, such as fatigue or myalgias, or who were asymptomatic with elevation of transaminases (without hepatic dysfunction). Patients were forwarded to the Reference Center for clinical evaluation and investigation.

### 2.2. Protocol

In the first step, high levels of CK (at least 1.5 times the standard values) were confirmed by two measures separated by one month and with an indication of exercise avoidance in the week before blood collection (exercise was allowed to increase until 30× CK levels) [13]. Afterward, systemic causes were excluded: medication use, drug abuse, endocrine causes, viral myositis, and rheumatologic diseases. The following step was the performance of a forearm test (with measurement of lactate, ammonia, and CK pre- and post-exercise). At this time, screening for Pompe disease was performed and a profile of acylcarnitines and organic acids was collected.

As for male patients with muscular hypertrophy, the first exam was a molecular study for DMD. Additionally, in some cases, a genetic panel or a muscle biopsy was conducted.

Depending on the initial clinical exam, muscle resonance imaging (MRI) or electromyography (EMG) was considered.

## 3. Results

A total of ten departments were visited, and eight of them enrolled patients.

Thirty-three patients were recruited: seventeen patients from the Centro Materno Infantil do Norte, five from Centro Hospitalar Tâmega e Sousa, four from Centro Hospitalar Trás-os-Montes Alto Douro, two from Hospital Senhora de Oliveira Guimarães, two from Centro Hospitalar entre o Douro e Vouga, two from Centro hospitalar Médio Ave, and one from Hospital de Braga.

**Figure 1:**
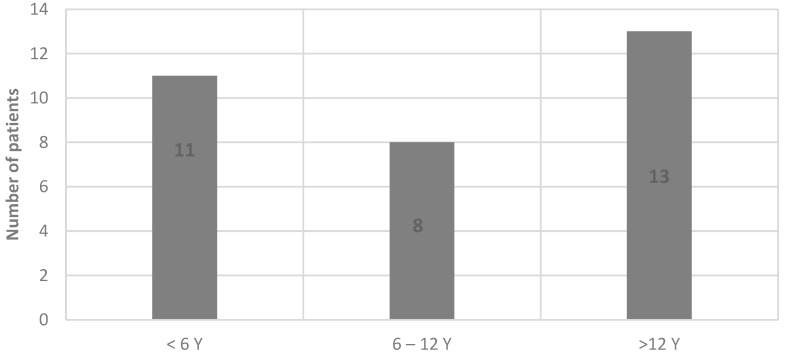
Distribution by age groups.

Among the total patients included in the study, one of them was excluded due to ordinary CK values. Most of the patients were males (N = 28). The average age of the patients was 7 years (ranging from 6 to 17 years). The most common secondary causes were infectious (viral myositis) and vigorous exercise. The age distribution is shown in Figure 1.

**Figure 2:**
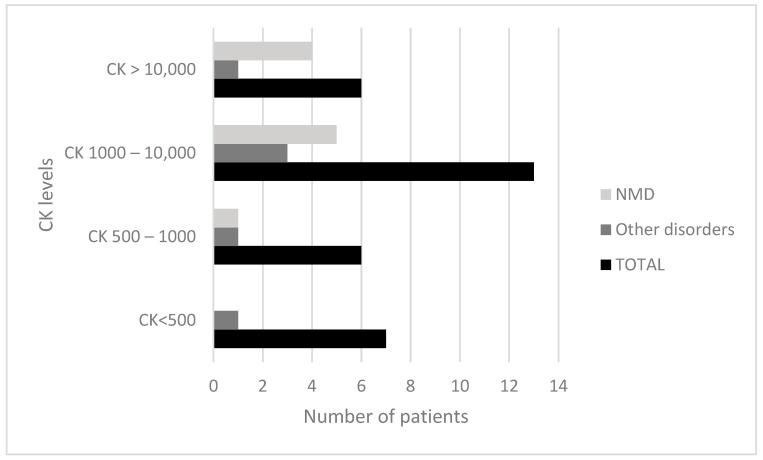
Correlation between CK levels and diagnostic test results. The total number corresponds to the overall number of patients with and without a diagnosis. CK: creatine phosphokinase.

The correlation between CK values and diagnoses is shown in Figure 2. A higher number of patients (N = 13) had CK levels between 1000 and 10,000 UI/L, followed by CK levels of <500 UI/L (N = 7), 500–1000 UI/L (N = 6), and >10,000 UI/L (N = 6).

All patients with CK < 500 UI/L, with posterior normalization, had typical muscular clinical examinations, and one of them was associated with isotretinoin treatment.

Of the patients with CK levels between 500 and 1000 UI/L, one had a Charcot–Marie–Tooth diagnostic (without an identified gene).

In the group of patients with CK levels between 1000 and 10,000 UI/L, muscular Becker dystrophy (BMD) (N = 4) and DMD (N = 1) diagnoses were identified.

Regarding patients with CK levels of >10,000 UI/L, one presented DMD, one gamma-sarcoglicanopathy, one CPT2, and one non-classified myopathy.

Of the patients with a CK > 500 UI/L, nine did not have a diagnosis and continued to present high levels of CK; therefore, they continued in follow-up.

**Figure 3:**
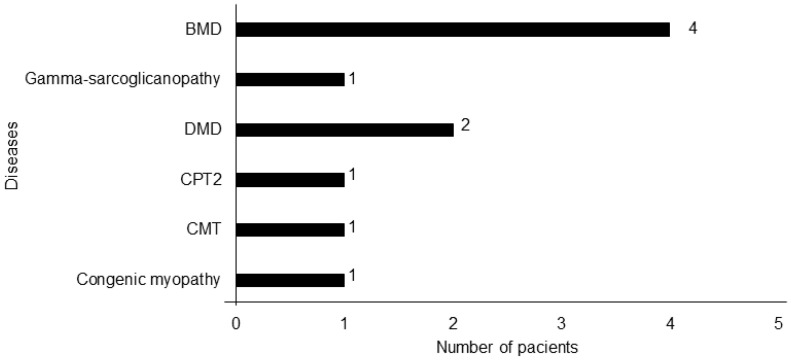
Neuromuscular disease diagnostics identified. CPT2: carnitine palmitoyl transferase 2 deficiency; CMT: Charcot–Marie–Tooth; DMD: Duchenne muscular dystrophy; BMD: muscular Becker dystrophy.

From the sweeping of 32 patients with elevated CK levels, 10 presented diagnoses of NMDs (31.25%) (Figure 3).

Fifteen of the patients with CK > 500 UI/L had a diagnosis: dystrophinopathies (N = 6), myositis (N = 5), gamma-sarcoglicanopathy (N = 1), carnitine palmitoyl transferase 2 deficiency (CPT2) (N = 1), non-classified myopathy (N = 1), and neuropathy (N = 1).

**Figure 4:**
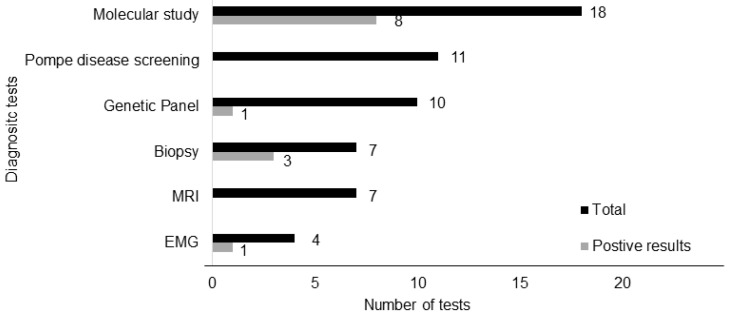
Auxiliary diagnostic tests.

The genetic study for dystrophy was the required test that presented higher positive results. A muscular biopsy was performed in seven patients, leading to three diagnostics: two patients without deletion or duplications for DMD/BMD had a dystrophic pattern and dystrophin irregularities; another one showed a myopathic pattern, with changes in the intern structure in a patient with congenital myopathy. In the case of the patient with CMT, the diagnosis was based on clinical findings (muscle weakness, reduced tendon reflexes, and sensory loss) associated with suggestive changes in nerve conduction studies and electromyography (EMG). The diagnostic tests are described in Figure 4.

## 4. Discussion

Innovative targeted treatments for NMDs can improve the course of the illness; however, diagnostic delay in these diseases can be a barrier. The irreversible damage that takes place before the diagnosis can significantly limit treatment efficacy [14]. Additionally, awareness about the importance of an early diagnosis of NMDs could optimize their treatment and improve the prognosis.

The analysis of CK levels in an early evaluation of children with developmental delays was recommended more than ten years ago [15]. Just recently, CK levels have been used in pilot studies in NBS (newborn screening) for diagnosis of Duchenne muscular dystrophy [10].

Innovative targeted treatments for NMDs can improve the course of the illness, whereas diagnostic delay in these diseases can be a barrier. The irreversible damage that occurs before the diagnosis can significantly limit treatment efficacy [14]. Awareness about the importance of an early diagnosis of an NMD could optimize the treatment and improve the prognosis.

In our study, we aim to evaluate paucisymptomatic or asymptomatic children with elevated transaminases, who have a higher risk of having an NMD. This was a pioneer study in Portugal, and there are a limited number of studies available in the literature. Most of them include follow-ups on patients from tertiary centers or patients’ family members with established diagnoses [16,17]. Moreover, it was possible to reach a diagnosis in half of the patients included in this study, in contrast to other published works, which show a diagnostic percentage of around 18% [18]. Furthermore, in our study, all pediatric hospital clinicians became aware of these disorders and the importance of CK measurement. All patients with NMD were followed by a multi-disciplinary team in their initial stage of disease.

Even if the most important role in the diagnosis of NMDs is played by clinical and subsequent examinations such as neurophysiological, histo-morphological, and genetic analyses, serum biomarkers may have a crucial role in the diagnosis and follow-up [19].

Herein, 31.25% of the patients presented diagnoses of NMDs. Other studies in the literature show much lower numbers, which can be explained by the CK levels or the characteristics of the population included in the study.

In the prospective study from Kodatsch and Stollberger, only 2% of the 100 recruited patients with elevated CKs were diagnosed with NMDs [20].

In a recent pilot study, Ricci et al. analyzed the feasibility of a screening program to identify children at high risk of NMDs within the first 30 months of life. The authors observed that, in the first-year feasibility study, after 10,032 visits to primary care pediatricians were conducted, twenty children screened positive for NMDs. Of these, four had elevated CK serum levels (20%) [21].

Similarly to the results observed in the literature, among the patients diagnosed in our study, dystrophinopathies outnumbered other types of muscular dystrophies (21%) [22]. One patient who was diagnosed with DMD had a treatable point mutation and an indication for ataluren. Additionally, even though not all patients with CK levels inferior to 500 IU/l were diagnosed with NMDs, clinical observation and a follow-up are necessary and recommended until complete and sustained normalization is achieved.

Although CK levels are an important parameter in the differential diagnosis of NMDs, they can also occur as an incidental finding without diagnostic significance and should always be evaluated in the context of clinical findings [23]. Moreover, some patients with a stationary clinical condition and absence of muscular symptoms can present hyperkalemia, which is not associated with any biological or genetic diagnosis of muscular disorders.

Screening programs in children with developmental delays have the potential to identify individuals with these rare but treatable conditions at an early stage and to reduce the time to start therapy, improving health, development, and life expectancy [24]. There has been a consensus among experts that these programs need to be implemented in order for the health system to be prepared for a rapid increase in transformative therapies [25,26].

This study has some limitations—it consists of a hospital-based population within the National Service (SNS), and only 8 of the 12 pediatric departments actively participated. Children from outpatient clinics and private hospitals were not included. Enrollment of children into pediatric studies or screenings remains challenging [27].

## 5. Conclusions

Many NMDs have a progressive and devastating impact on the patients’ lives and their families. There has been a continuous amelioration in the care of these patients. In some of them, there are therapeutic measures that can be implemented with clear benefits for survival and quality of life. New treatments are expected to be available in the coming years. An early diagnosis is crucial to prevent disease progression and reduce symptoms. CK level determination is an inexpensive, simple, minimally invasive, and excellent screening tool in patients with suspected muscle disorders. A careful history, examination findings, and CK values can provide a road map to arrive at a specific etiologic diagnosis with other additional tests.

## Data Availability

The original contributions presented in the study are included in the article; further inquiries can be directed to the corresponding author.
